# Thioredoxin-A is a virulence factor and mediator of the type IV pilus system in *Acinetobacter baumannii*

**DOI:** 10.1371/journal.pone.0218505

**Published:** 2019-07-02

**Authors:** Holly C. May, Jieh-Juen Yu, Hao Zhang, Yufeng Wang, Andrew P. Cap, James P. Chambers, M. Neal Guentzel, Bernard P. Arulanandam

**Affiliations:** 1 South Texas Center for Emerging Infectious Disease and Department of Biology, University of Texas at San Antonio, San Antonio, Texas, United States of America; 2 Coagulation and Blood Research Program, US Army Institute for Surgical Research, JBSA Fort Sam Houston, San Antonio, Texas, United States of America; University of Kansas Medical Center, UNITED STATES

## Abstract

The Gram-negative pathogen, *Acinetobacter baumannii* has emerged as a global nosocomial health threat affecting the majority of hospitals in the U.S. and abroad. The redox protein thioredoxin has been shown to play several roles in modulation of cellular functions affecting various virulence factors in Gram-negative pathogens. This study aims to explore the role of thioredoxin-A protein (TrxA) in *A*. *baumannii* virulence. We determined that deletion of the *TrxA* gene did not significantly affect resistance to environmental stressors such as temperature, salt, and pH. However, TrxA was critical for survival in the presence of elevated levels of hydrogen peroxide. Lack of TrxA was associated with decreased expression of type IV pili related genes and an inability to undergo normal twitching motility. Interestingly, the TrxA-null mutant was able to form biofilms better than the wildtype (WT) and was observed to be significantly less virulent than the WT in a pulmonary infection model. These results are supportive of thioredoxin playing a key role in *A*. *baumannii* virulence.

## Introduction

*Acinetobacter baumannii*, an aerobic, Gram-negative, coccobacillus, has emerged as a major global health threat [1, 2]. Its ability to tolerate desiccation as well as increased multi-drug resistance (MDR) have made this organism an important emerging nosocomial pathogen [3]. In a 2010 study, greater than 50% of *A*. *baumannii* clinical isolates were classified as MDR, *i*.*e*., resistant to greater than 3 classes of antibiotics [4]. Additionally, *A*. *baumannii* occupies the top spot on the World Health Organization’s recent list of global bacterial pathogens for which new treatment regimens are critically needed [5]. Critically ill patients, *i*.*e*., those in intensive care units (ICU), are at the highest risk for *A*. *baumannii* infections, giving rise to pneumonia, meningitis, septicemia, urinary tract, and wound infection [6–9]. Of the above, hospital-acquired pneumonia is the most severe clinical presentation associated with *A*. *baumannii* [6] followed by bloodstream infections in ICU patients [10]. *A*. *baumannii* nosocomial infections lead to increased hospitalization costs [11], and are associated with mortality rates greater than 50% [12].

Thioredoxin-A (Trx1/TrxA) is a member of the thioredoxin protein superfamily, and is typically a 12 kDa thiol-disulfide oxidoreductase with a multitude of functions including reducing protein disulfides [13]. Previous research has indicated that thioredoxin can also play a role in bacterial virulence. In *Listeria monocytogenes*, thioredoxin was observed to be critically important for proper functioning of key regulators in motility and gene transcription [14]. *Helicobacter pylori* has been shown to utilize secreted thioredoxin for reduction of mucin, enhancing the ability of the microorganism to make contact with the epithelial surface [15]. Furthermore, *Mycobacterium tuberculosis* lacking a functioning thioredoxin system was cleared more readily in a pulmonary infection model [16]. Lastly, a thioredoxin mutant of *Francisella novicida* was observed to have decreased virulence with increased host survival in a murine inhalation infection model [17].

Previously, we observed that *A*. *baumannii* can evade host mucosal immune defenses by TrxA-mediated dissociation of the secretory component from sIgA. Using a TrxA-null mutant (ΔtrxA) generated from MDR *A*. *baumannii* clinical isolate 79 (Ci79) by homologous recombination, we demonstrated that TrxA is a key virulence factor involved in bacterial colonization of the gastrointestinal (GI) tract [18]. Furthermore, lack of thioredoxin leads to a 100-fold increase in the 50% lethal dose (LD_50_) following systemic challenge, and mice immunized with live ΔtrxA mutant were able to survive subsequent intraperitoneal challenges of 10 times the WT strain LD_50_ with no observable pathology in target organs [19].

The purpose of this study was to further characterize *A*. *baumannii* thioredoxin function utilizing the ΔtrxA mutant. Data reported here further support thioredoxin playing a crucial role in *A*. *baumannii* virulence.

## Materials and methods

### Ethics statement

All experiments utilizing animals were performed in compliance with the Animal Welfare Act and the recommendation in the Guide for the Care and Use of Laboratory Animals of the National Institutes of Health under the approved protocols MU070 and MURA005 in accordance with guidelines set forth by the University of Texas at San Antonio Institutional Animal Care and Use Committee (IACUC). Animals were euthanized by CO_2_ inhalation followed by cervical dislocation when the experimental endpoints were reached.

### Bacterial strains

*Acinetobacter baumannii* clinical isolate 79 (Ci79) was obtained by the San Antonio Military Medical Center (SAMMC; Fort Sam Houston, San Antonio, TX) from injured military personnel, and provided by Dr. James Jorgensen (The University of Texas Health Science Center at San Antonio, San Antonio, TX). The ΔtrxA mutant strain was generated from WT Ci79 strain via homologous recombination with addition of an erythromycin resistance gene for selection as previously reported [18]. The complemented strain ΔtrxA^C^ (designated as Comp in this report) was complemented with the Ci77 strain TrxA which shares 100% amino acid sequence homology to Ci79, with the added presence of a synonymous SNP resulting in loss of a *Sal*I restriction site [18]. Unless otherwise stated, all bacteria were grown from frozen stocks on Luria-Bertani (LB) agar plates and incubated at 37 ^0^C for 24 hours before subculturing in LB broth. Overnight liquid bacterial cultures were diluted 1:100 and grown to mid-log phase. Growth curves for strains Ci79, ΔtrxA, and Comp were individually generated for determination of bacterial CFU/mL based on optical density measured at 600 nm. Inoculums were determined by plating serial dilutions on LB agar plates.

### Mice

All animal experiments were performed using 4–6 week-old female C57BL/6 mice purchased from Jackson Laboratories (Bar Harbor, ME). Animals were housed at the University of Texas at San Antonio animal facility which is accredited by Association for Assessment and Accreditation of Laboratory Animal Care International (AAALAC). All experiments were performed in accordance with the guidelines set forth by the Institutional Animal Care and Use Committee (IACUC). Mice challenged with *A*. *baumannii* were monitored and weighed daily. When mouse mobility was compromised, MediGel was placed in the cages so that all animals had direct source of fluids. Mice were monitored and weighed daily. When animals became symptomatic (such as inactivity, sunken eyes, hunched posture piloerection/matted fur), they were monitored twice daily not more than 14 hours apart. Any animal that was clearly terminal as indicated by lack of activity, difficulty in breathing, ruffled fur persisting for 24 hours or dramatic loss of body weight greater than 20% were euthanized in a closed chamber with CO_2_ (no response to vigorous rear toe pinch) followed by cervical dislocation. There was no mouse died before meeting criteria for humane endpoint euthanization during this study.

### Transmission electron microscopy

Bacteria were grown to an OD_600_ of 0.8. Cultures were pelleted by centrifugation (3,500 x g for 10 minutes) and resuspended in PBS. Samples were placed on a Formvar/carbon coated grid, stained with 2% (w/v) uranyl acetate, and examined using a JEM-1400 Plus Transmission Electron Microscope (Electron Microscopy Lab, Department of Pathology, The University of Texas Health Science Center at San Antonio, San Antonio, Tx).

### Transcriptome analysis using RNA-seq

Bacteria were grown to mid-log phase for RNA isolation using a PureLink RNA Mini Kit (Ambion, Thermo Fisher Scientfic, Waltham, MA). RNA was enriched for mRNA by removal of rRNA using a MICROBExpress Kit (Ambion). Library preparation and RNA-seq analysis were carried out on an Illumina HiSeq 3000 instrument with 50bp single read sequencing (The University of Texas Health Science Center at San Antonio Genome Sequencing Facility). Sequencing reads were preprocessed using the Trim Galore program [20] with default parameters to remove adapters and low-quality sequences. Reads were aligned to *Acinetobacter baumannii* strain Ci79 reference genome ASM51663v2 (GenBank assembly accession: GCA_000516635.2) using HISAT2 with default settings [21], and counted using the featureCounts program [22]. After eliminating low expression genes, *i*.*e*., < 1 count per million (CPM) in three or more samples across the data set, raw expression values were normalized using the edgeR Program’s calcNormFactors function [23]. Differentially expressed genes (DEGs) were determined by quasi-likelihood F-test using the edgeR program’s glmQLFit function. Genes exhibiting an FDR-adjusted P-value (≤0.05), and fold-change ≥2 or ≤-2 were considered to be differentially expressed in this study. Heatmaps were generated with Prism Graphpad and the volcano plot was generated using R functions.

### qRT-PCR

Bacteria were grown to mid-log phase for RNA isolation, DNase treatment, and RNA clean-up using a PureLink RNA Mini Kit (Ambion, Thermo Fisher Scientific, Waltham, MA). RNA was converted to cDNA with the iScript cDNA Synthesis Kit per manufacture instructions (Bio-Rad, Hercules, CA). qRT-PCR was conducted using the SsoAdvanced Universal SYBR Green Kit (Bio-Rad, Hercules, CA) with *A*. *baumannii* Ci79 gene specific primer pairs targeted to ComB, ComO, PilT, PilV, and GAPDH (Intergrated DNA Technologies, Coralville, IA) [24]. qRT-PCR was run on the Bio-Rad CFX96 Real-Time System and the CFX Maestro software (Bio-Rad, Hercules, CA). qRT-PCR data were analyzed using the ΔΔCq method [25] to determine the overall relative gene expression in ΔtrxA compared with WT from 3 independent studies. The average ct of each gene from triplicates of WT and mutant samples was used for ΔΔCq calculation. In each study, relative gene expression in ΔtrxA was compared to WT as value of 1 after normalization with the reference gene GAPDH.

### Motility assay

Bacterial twitching motility was investigated using a modified protocol reported by Wood [26]. Briefly, overnight cultures were collected using a sterile pipette tip, and stabbed through the 0.3% (w/v) agar LB layer to the agar/plate interface. Plates were grown right-side up for 48 hours followed by soft agar removal, staining with 0.1% (w/v) crystal violet for 10 minutes, washing (one time), and measurement of growth diameter.

### Biofilm assay

Biofilm formation was determined using a modification of the method of O’Toole [27]. Briefly, bacteria were streaked onto an LB plate, grown for 24 hours, subcultured into LB broth, and grown in a static incubator at 37 ^o^C overnight. Turbidity (OD_600_) was adjusted between strains, diluted 1:100 into fresh LB broth, transferred to either a 96-well plate or polystyrene tubes, and incubated for 24 hours at 37 ^o^C. Liquid was aspirated, and the plate or tube was washed once with PBS. Crystal violet (0.1%) was added and allowed to stand for 10 minutes followed by aspiration of dye, and two washings with PBS. Biofilm formation was quantified in the 96-well plate format by addition of 25% (v/v) acetic acid to each well, allowing to stand for 5 minutes, and determination of absorbance at 550 nm.

### Congo red binding assay

Congo Red binding was determined by suspending bacteria in PBS to an OD_600_ of 1.0 and mixing with 100 ng/mL Congo Red at 37 ^o^C for 15 minutes. Bacteria were centrifuged (3,500 x g for 10 minutes) and OD_480_ values compared to Congo Red alone in PBS. Calculation of percent bound Congo Red was determined using the formula: % CR bound = [1- (remaining Congo Red in bacterial supernatant/Congo Red in PBS)] x 100.

### Environmental stressor growth curves

Bacterial growth curves were carried out in 96-well plates. Overnight cultures were diluted 1:100. Bacteria were grown at different temperatures (30, 37, or 45 ^o^C) or at 37 ^o^C in the presence of different concentrations of salt (added to the already present 5g/L salt present in the media) (0%, 2.2%, and 4%), differing pH (5.5, 7.0, and 9.0, adjusted with NaOH or HCl), or percentage hydrogen peroxide (0%, 0.15%, and 0.3%). OD_600_ was determined every 2 hours for 8 hours, and subsequently at 12 and 24 hours.

### Pulmonary infection model

Mice were anesthetized by isoflurane inhalation, and 1 x 10^8^ CFU/mouse (unless otherwise indicated) in PBS was administered in 50 μL volumes to the lung by oropharyngeal aspiration [28]. Animals were allowed to recover before being returned to their cage. For bacterial burden assessment, mice were euthanized at 24 hours *post* challenge. Blood was collected via submandibular bleeding. Cervical lymph nodes, lungs, kidneys, liver, and spleen tissues were collected and placed in 1 or 5 mL (liver only) PBS. Organs were homogenized using a rotor stator mechanical homogenizer, and serial dilutions plated on LB agar to enumerate bacterial burdens. For survival, mice were observed and weighed daily for seven days following infection, and then monitored for 14 days to ensure that no latent effects from the infection were seen. For pathology assessment, immediately following euthanization, PBS containing heparin (20 units/mL) was immediately perfused through the heart followed by perfusion of 4% (v/v) buffered formalin solution (BFS). Lungs were removed and placed in 4% BFS for 24 hours prior to being transferred to 70% (v/v) ethanol. Paraffin blocks were prepared in the Department of Pathology, The University of Texas Health Science Center at San Antonio. Blocks were cut, stained with H&E, and pathology assessed via brightfield microscopy. Radial alveolar count (RAC) values were determined as previously described [29, 30]. Briefly, a perpendicular line was drawn from the respiratory bronchiole to either the pleura or the nearest connective tissue septum, and the number of bisected alveoli was counted.

### Statistics

Statistical differences were assessed by two-way ANOVA with Holm-Sidak correction for multiple comparisons. qRT-PCR significance was determined with unpaired T test comparing the normalized expression of the mutant to the WT. Survival statistical differences between challenge groups were determined using the Mantel-Cox log rank test. All statistics were performed using GraphPad Prism statistical software (San Diego, California).

## Results

### Thioredoxin is necessary to maintain normal cell surface

**Fig 1:**
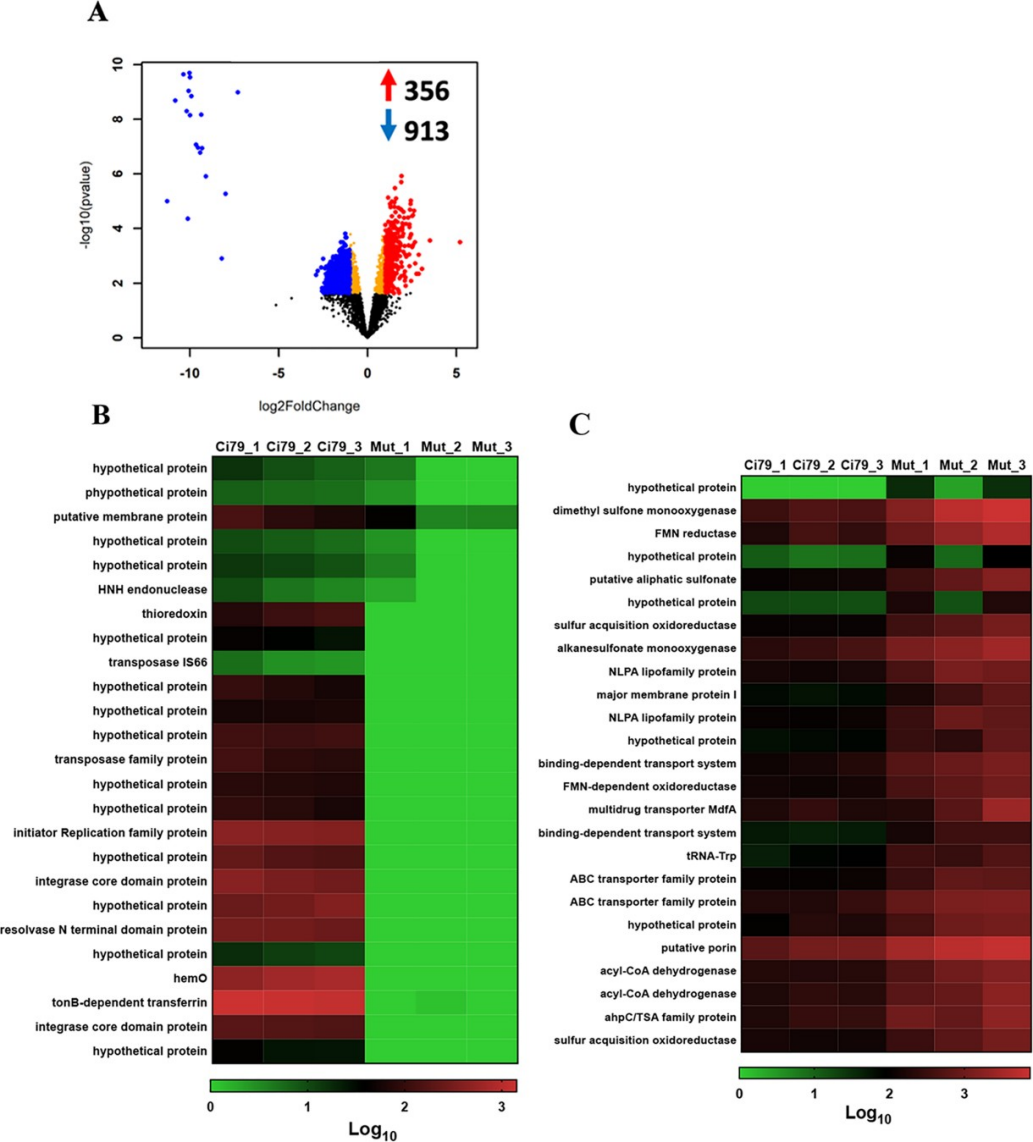
Comparison of gene expression in WT and trxA mutant strains. Assessment of gene expression was determined in triplicate by RNA-seq analysis. (A) Volcano plot. (B) Heatmap of mutant down-regulated genes. (C) Heatmap of mutant up-regulated genes.

Differences in WT and ΔtrxA global gene expression was evaluated by RNA-seq analysis. RNA-seq data were submitted to Gene Expression Omnibus and can be accessed using the accession number GSE125017. In total, 1,269 significant gene expression differences were observed with up-regulation of 356 genes and down-regulation of 913 genes in the mutant (Fig 1A). Many of the genes are annotated as hypothetical and, as such, have no known function(s). Due to the high number of hits, heatmaps are shown for only the top 25 down- (Fig 1B) and up-regulated genes (Fig 1C). A list of the fold-changes for the top 50 up and down regulated genes is listed in S1 Table.

**Fig 2:**
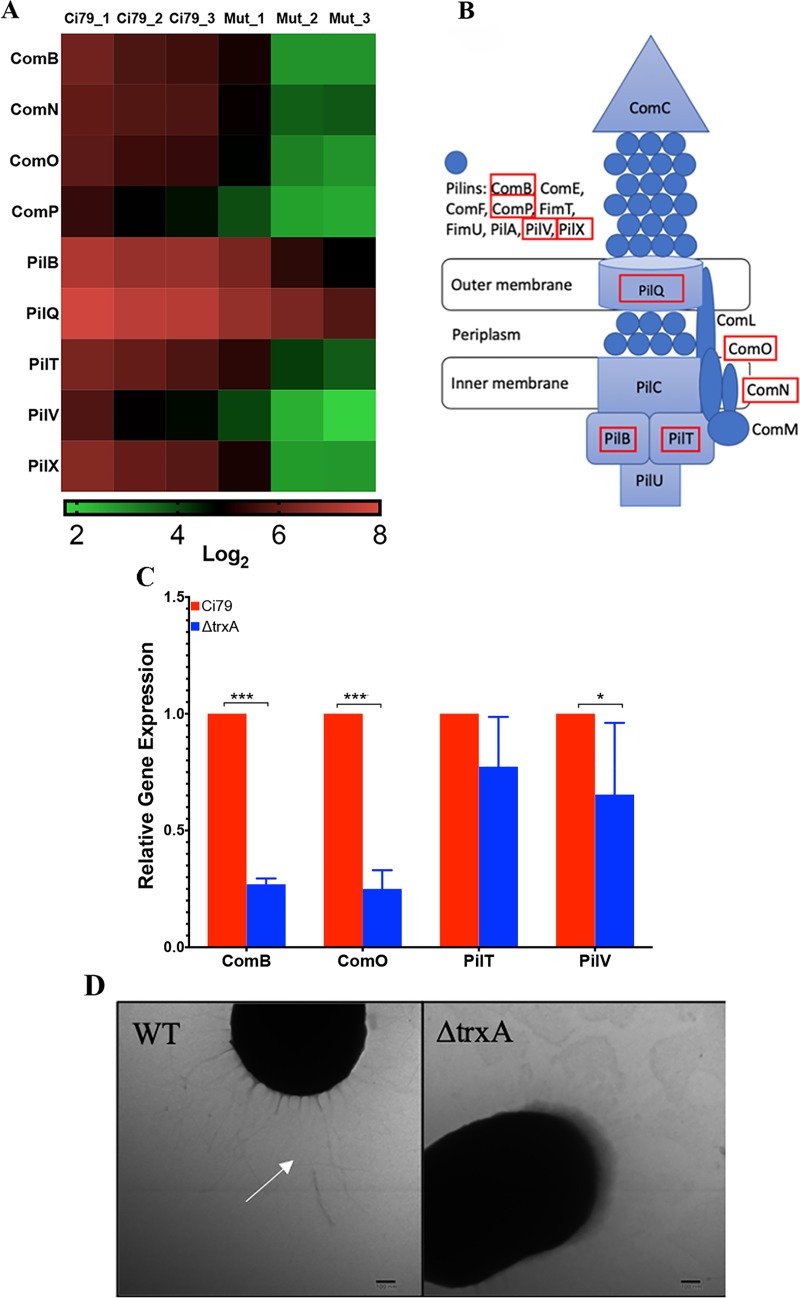
RNA-seq analysis reveals a crucial role for thioredoxin in type IV pili production. (A) Heatmap of differentially regulated genes associated with the type IV pili (T4P). (B) Schematic of the Gram-negative T4P with red boxes indicating the genes downregulated in the mutant. (C) qRT-PCR of select T4P genes. Error bars represent SD. Statistical differences were determined by unpaired T-test correction (***P < 0.001, *P < 0.05). (D) TEM images of cell surface with white arrow indicating pili.

One notable system with many gene product components down-regulated in the mutant was the type IV pili (T4P). Specifically, down-regulation was seen in ComB (M212_3777), ComN (M212_3801), ComO (M212_3800), ComP (M212_3799), PilB (M212_0349), PilQ (M212_3798), PilT (M212_1027), PilV (M212_3778), and PilX (M212_3776) (Fig 2A and 2B). Annotated gene names are listed in parentheses. Down-regulation of genes associated with the T4P in the mutant was further assessed by qRT-PCR using gene-specific primer sets (Table 1) for ComB, ComO, PilT, and PilV. As shown in Fig 2C, ComB, ComO, and PilV were found to be significantly down-regulated in the mutant strain, while PilT did not show a significant difference in expression between WT and ΔtrxA. Furthermore, TEM analysis of the bacterial cells revealed a visible loss of pili on the cell surface of ΔtrxA (Fig 2D).

**Table 1:** Primer sequences for qRT-PCR.

Name	Sequence (5’-3’)	Description
ComB_Fwd	CCACCCGATACTGAGGAATTG	ComB Forward Primer
ComB_Rev	TTAGGAGATGCAGGGCAAATTA	ComB Reverse Primer
ComO_Fwd	TCTTTAGCTGGCGATACATCAA	ComO Forward Primer
ComO_Rev	GGGACTATCACGCATTTGGA	ComO Reverese Primer
PilT_Fwd	CAAACACCAAGTGACCTGTTTC	PilT Forward Primer
PilT_Rev	CTGCGTGAAGACCCAGATATT	PilT Reverse Primer
PilV_Fwd	AGTTGGGAGGCAGTACATAGA	PilV Forward Primer
Pilv_Rev	TCAAGCTAGCCAGTATCCTACA	PilV Reverese Primer
GAPDH_Fwd	TCATGTACGACACAGGCTTTAG	GAPDH Forward Primer
GAPDH_Rv	CCGCATGAATTTCGGTCATAAG	GAPDH Reverse Primer

**Fig 3:**
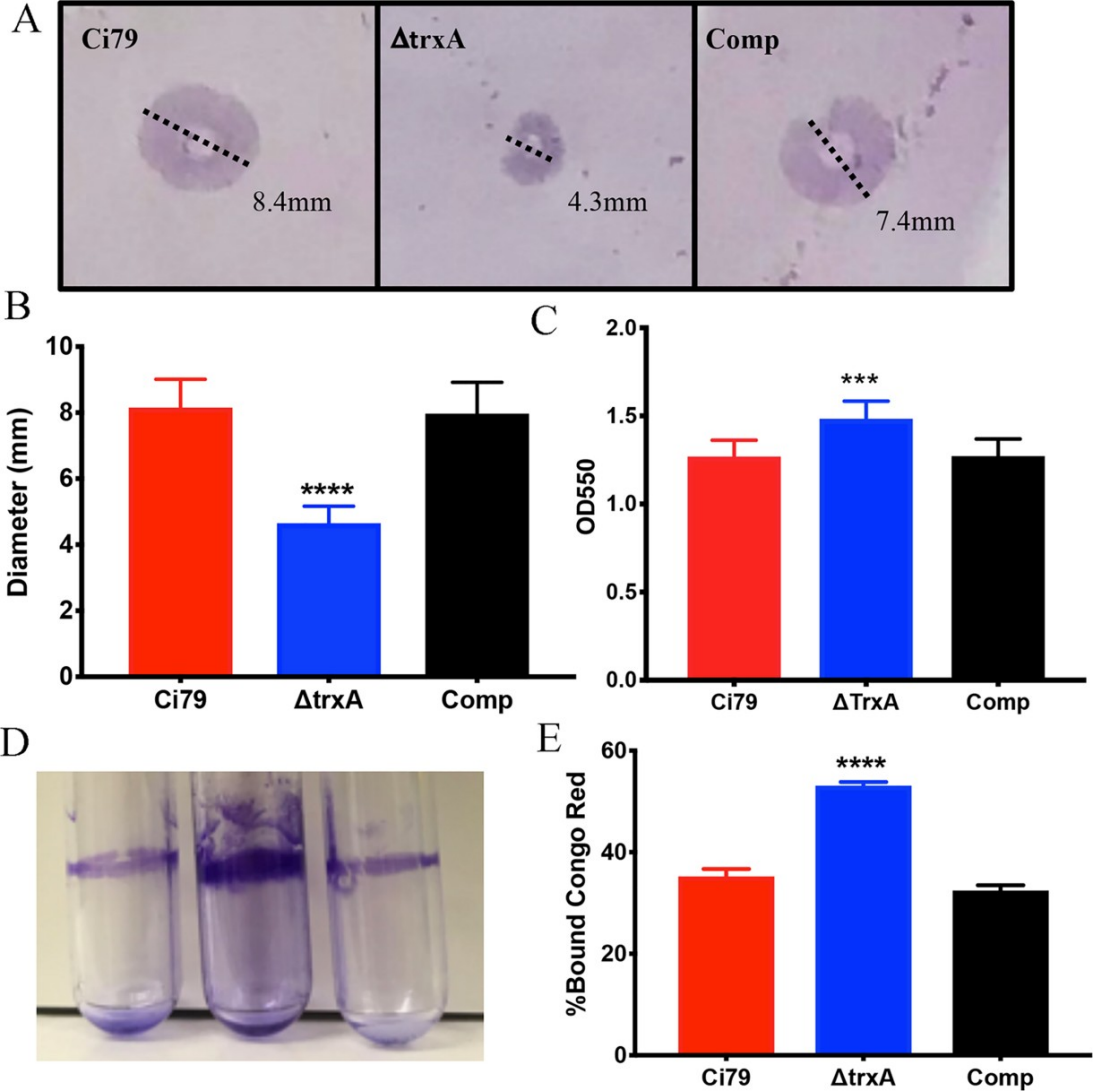
Comparison of growth characteristics and phenotype of wild type (Ci79), trxA mutant, and complemented bacterial strains. Twitching motility was measured by growing bacteria in a 0.3% LB agar plate that had been inoculated at the agar/plate interface. (A) Representative images depicting motility measurement, and (B) overall spreading distance (mean ± SD). Biofilm was measured by growing bacteria for 24 hrs in a static incubator at 37 ^o^C. (C) Quantitation of biofilm formation in 96 well format. (D) Visual comparison of biofilm formation in polystyrene tubes. Congo Red binding (E) was assessed on mid-log phase bacteria. Error bars represent SD. Bar charts represent one set of data from 3 independent studies, each consist of 6 samples for each bacterial strain. Statistical differences were determined by two-way ANOVA with Holm-Sidak correction for multiple comparisons (***P < 0.001, ****P < 0.0001).

Twitching motility and biofilm formation have been associated with T4P and virulence in *Acinetobacter* and certain other Gram-negative organisms [31–33]. Because pili are necessary for twitching motility and promote biofilm formation, differences in these functions in WT and ΔtrxA mutant were investigated. As shown in Fig 3A and 3B, ΔtrxA mutant motility was approximately 50% reduced compared to WT and Comp strains. Counterintuitively, measurement of biofilm formation indicated the ΔtrxA mutant to form a more dense biofilm than that of both WT and Comp strains (Fig 3C and 3D). Observed differences in biofilm thickness and formation were suggestive of cell surface changes prompting assessment of cell surface hydrophobicity by Congo Red binding assay. The percentage of Congo Red bound to the ΔtrxA mutant was significantly greater than that of the WT and Comp (Fig 3E) indicating that TrxA deficiency resulted in an increase of bacterial surface hydrophobicity.

### Lack of thioredoxin leads to a decreased resistance to hydrogen peroxide exposure

Resistance to common Gram-negative antibiotics was analyzed to determine whether deletion of *TrxA* gene affects antibiotic resistance. The WT was observed to be resistant to all antibiotics tested with the exception of moderate susceptibility to cefepime and ceftazidime in contrast to the ΔtrxA mutant strain which was completely resistant to ceftazidime (S2 Table). In order to determine if thioredoxin is required for survival in response to various stressors, the ΔtrxA mutant strain was subjected to different growth temperatures, and varying salt, pH, and hydrogen peroxide concentrations at 37 ^0^C. Bacterial growth at 30 and 45 ^o^C, when compared to growth at 37 ^0^C, was reduced for all three strains. As expected, ΔtrxA exhibited a slower growth rate at all temperatures [18]. However, no difference was noted in the rate of growth between strains, indicating that thioredoxin does not play a role in growth response to temperature (S1A Fig). Increased salt, *i*.*e*., 2.2 and 4.4% added to LB media containing 1.0% (w/v) NaCl, was shown to slow growth in a dose-dependent manner in all strains. Although the ΔtrxA mutant strain did exhibit significant but delayed decreased growth rate, no significant differential effect was observed in response of the three strains to excess salt (S1B Fig). Additionally, little difference in the growth rate of individual strains as a function of decreasing H^+^ ion concentration, *i*.*e*., pH 5.5, 7.0, and 9.0 was observed (S1C Fig).

**Fig 4:**
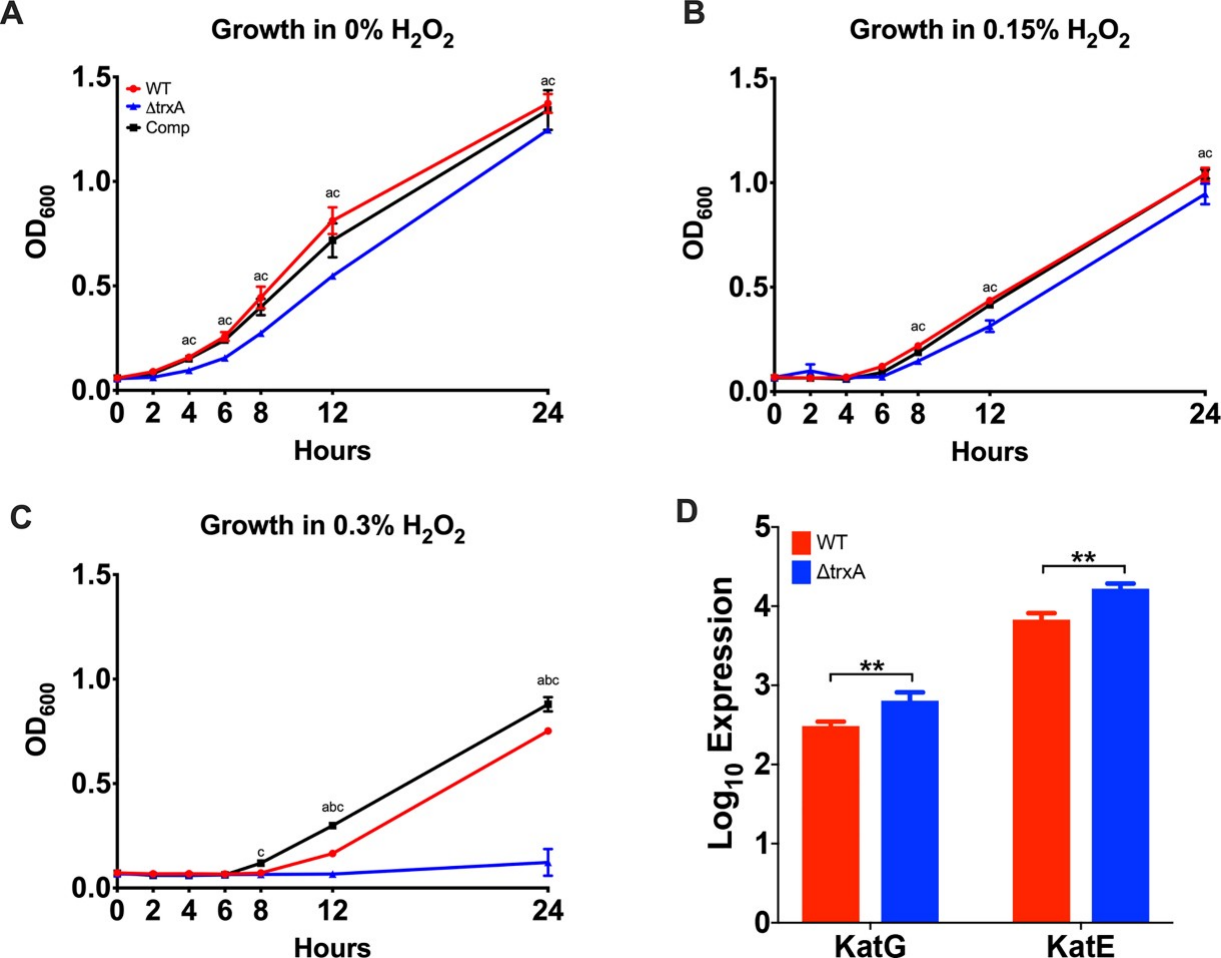
Comparison of the effects of hydrogen peroxide on WT, ΔtrxA, and Comp bacterial strains. (A-C) Growth curve of bacteria grown in the absence and presence of hydrogen peroxide. Representative of 3 independent experiments, n = 6 per bacterial strain. a = significant between groups 1&2, b = significant between groups 1&3, c = significant between groups 2&3. Statistical differences were determined by two-way ANOVA with Holm-Sidak correction (P < 0.05). (D) Transcription analysis of catalase genes, KatG and KatE, by RNA-seq. **, P < 0.005.

Because TrxA is metabolically involved in handling of reactive oxygen species, *e*.*g*., formation of peroxides, the effect of increased levels of hydrogen peroxide, *i*.*e*., 0.15 and 0.3% (v/v) on growth was determined (Fig 4). As shown in Fig 4B, all three strains exhibited decreased growth in the presence 0.15% hydrogen peroxide supplemented medium. However, WT and Comp strains were able to overcome exposure to 0.3% hydrogen peroxide and resumed growth 8 hours *post* exposure, in distinct contrast to the ΔtrxA mutant which was unable to recover even at 24 hours *post* exposure (Fig 4C). It has been shown that catalases also play an important role in hydrogen peroxide resistance. However, KatG (M212_0431) and KatE (M212_1650), two catalase genes responsible for hydrogen peroxide resistance in *A*. *baumannii* [34], were found to be upregulated in the mutant strain in our transcriptomic analysis, this upregulation was not enough to overcome the loss of thioredoxin (Fig 4D).

### ΔtrxA is less virulent in a pulmonary model of infection

**Fig 5:**
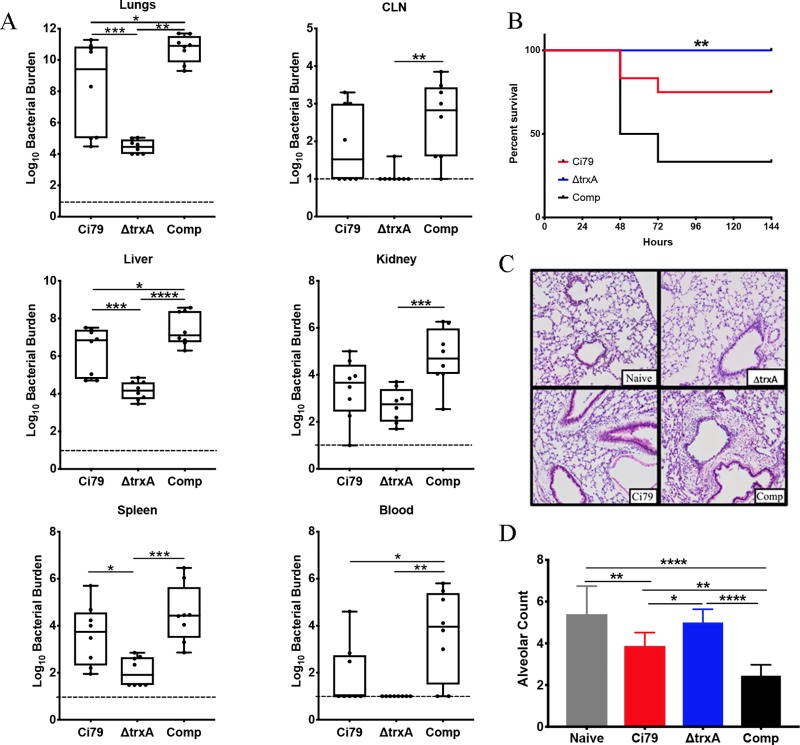
Comparison of pathogenesis of *A*. *baumannii* wild type (Ci79), trxA mutant, and complemented bacterial strains in a mouse pulmonary model. Mice were infected with bacteria (1x10^8^ CFU/mouse) via oropharyngeal aspiration. (A) At 24 hours, mice (n = 6 per group) were sacrificed and blood and organs were collected for bacterial burden measurement. Representative of 3 independent studies. (B) Survival of bacterial-challenged mice (n = 6 per group) was monitored for 7 days. **, P = 0.0012 (Mantel-Cox log rank test) between ΔtrxA and Comp. No statistical significance was found between ΔtrxA and WT. Representative of 2 independent studies. (C) For pathology, mice lungs were removed at 24 hours post challenge and fixed and embedded before sectioning and staining with H&E. Pathology was limited in all cases, although increased infiltration and thickening of alveolar walls was noted in WT and Comp. (D) To quantify pathology, differences in the number of alveolar spaces was determined by using radial alveolar counts. Error bars represent SD. Statistical differences were determined by two-way ANOVA with Holm- Sidak correction for multiple comparisons for bacterial burden (A) and alveolar space (D), ****, P < 0.0001; ***, P < 0.0005; **, P < 0.005; *, P < 0.05.

Pneumonia is one of the leading causes of mortality by *Acinetobacter* [35]. We applied a mouse model of respiratory bacterial challenge to assess the importance of TrxA as an *A*. *baumannii* virulence factor and found that deletion of the *TrxA* gene resulted in decreased bacterial burden at the challenge site as well as reduced dissemination to other target organs 24 hours *post* aspiration. Specifically, the ΔtrxA mutant exhibited significantly decreased organ burdens in the lungs, liver, and spleen compared to WT with even more pronounced reduced burdens compared to the respective Comp strain tissues (Fig 5A). All animals survived pulmonary ΔtrxA mutant (1x10^8^ CFU) challenge while 50 and 75% survived Comp and WT bacterial challenge, respectively (Fig 5B). To further investigate host response to infection, lung pathology was assessed 24 hours *post* challenge. With the exception of somewhat greater infiltration of WT and Comp strains, no major difference in lung pathology was observed (Fig 5C). An additional assessment of lung pathology, *i*.*e*., the radial alveolar count (RAC) was carried out. As shown in Fig 5D, WT and Comp infected lung tissues exhibited significantly lower RAC values compared to untreated (naïve) and ΔtrxA mutant infected lungs, while RAC values of ΔtrxA mutant infected lungs were not significantly different than untreated naïve controls (Fig 5D). Thus, alteration in alveolar structure and loss of lung parenchyma in mice infected with WT or Comp indicates increased pulmonary damage compared to naive and ΔtrxA mutant challenge.

## Discussion

Multi-drug resistant *Acinetobacter baumannii* (MDR-Ab) is rapidly becoming a significant concern worldwide for both civilian and military personnel. In 2013, the CDC classified MDR-Ab as one of the greatest emerging health threats in the United States due to the prevalence of emergent multi-drug resistance [36] while the World Health Organization has recently listed *Acinetobacter baumannii* as the number one global “Priority 1: Critical” pathogen for research and development of new therapeutics, *i*.*e*., antibiotics for treatment [5]. Furthermore, with continued use of antibiotics, multi-, extensively-, and pan-drug resistant strains have begun to emerge [37] making treatment problematic and necessitating development of alternative treatment regimens.

The bacterial thioredoxin system is coded for by three genes, a thioredoxin reductase, and thioredoxin-A and C genes. Thioredoxin-A (TrxA) is highly expressed in contrast to the less expressed thioredoxin-C (TrxC) which contains two additional thioredoxin fold motifs [38, 39]. While TrxC does appear to be redundant in light of the highly expressed TrxA, the ability of a bacterium to survive without an operating thioredoxin system is generally dependent upon a secondary redox system such as the glutathione system [40]. Gram-negative bacteria tend to contain both thioredoxin and glutathione systems while Gram-positive bacteria tend to have only a thioredoxin system making the production of thioredoxin knockout mutants in Gram-positive bacteria difficult as a redox system is necessary for viability [13].

In this study, we investigated the importance of thioredoxin in *A*. *baumannii* virulence. Loss of thioredoxin had little impact on susceptibility to common antibiotics used against Gram negative infections. However, comparative gene expression analyses by RNA-seq and qRT-PCR with supporting morphological TEM indicate decreased formation of Type 4 pili in ΔtrxA. In subsequent assessment of pili-associated phenotypes, we found that TrxA deficiency also led to decreased twitching motility and increased biofilm formation in *A*. *baumannii*. Thioredoxin has previously been linked to bacterial motility. Cheng *et al*. observed that 1) TrxA is necessary for formation of flagella (Type III secretion) in *Listeria monocytogenes*, and 2) a thioredoxin knock-out was completely non-motile [14]. Additionally, *A*. *baumannii* lacking the redox protein superoxide dismutase (SOD) was found to be deficient in motility [41]. Although, alteration of T4P assembly by TrxA has not been reported, Reardon-Robinson *et al*. have shown that deletion of a thiol-disulfide oxidoreductase (MdbA), whose structure exhibits a conserved thioredoxin-like domain, prevented the assembly of shaft pilin SpaA possibly by reducing the disulfide bound required for forming the pilus structure in a Gram-positive bacterium [42].

Interestingly, cell surface changes can be correlated with changes in hydrophobicity which have, in turn, been associated with initial adherence. The increased ability to form biofilms could be explained by increased adherence to the plastic surface at 24 hours while motility, measured at 48 hours, requires secondary processes subsequent to adherence. Congo Red binding has previously been used to measure cell surface hydrophobicity, although this is often done assuming that the Congo Red is binding to bacterial amyloids [43]. Although *Acinetobacter* has not been shown to produce curli, the most common type of bacterial amyloid, the percentage of Congo Red bound to the ΔtrxA mutant was significantly greater than that of the WT. This lends credence to the hypothesis that the increased biofilm formation observed in the thioredoxin mutant is related to changes in cell surface hydrophobicity. Additional studies are required to investigate other genomic differences noted in the RNA-seq data of ΔtrxA such as a down-regulation of Type VI secretion system genes and genes related to iron uptake and metabolism, as well as the up-regulation in many genes associated with ABC transport systems.

Although phenotypic differences were present, little difference in the ability of the ΔtrxA mutant to survive environmental stressors was observed. In all three strains, growth was slower at 30 and 45 ^0^C with little to no difference between the respective strains at a normal incubation temperature, *i*.*e*., 37 ^o^C. Similar growth curves were observed in the presence of increased salt concentration with growth being slowed moderately in the presence of 2.2% added NaCl and significantly more in the presence of 4.4% NaCl. Changes in H^+^ ion concentration, *i*.*e*., media pH, had little effect on growth in all cases examined. However, differences were observed in the ability of the ΔtrxA mutant to grow in the presence of hydrogen peroxide. While the WT and Comp strains were able to recover growth in 0.3% hydrogen peroxide, the ΔtrxA mutant was not. While this could potentially have been caused by a loss of catalase gene expression, the RNA-seq expression levels of KatG and KatE were found to be overexpressed in the mutant, indicating that the loss of thioredoxin was mediating the loss of hydrogen peroxide resistance in the mutant, likely due to the ability of thioredoxin to protect the cell from reactive oxygen species through reduction of disulfide bonds.

Pathogenicity was investigated using a murine pulmonary infection model. The ΔtrxA mutant challenged mice were observed to have lower organ bacterial burdens *post* challenge, and increased survival. We have previously shown that *in vitro*, there is no difference in protein expression in thioredoxin between the WT and Comp strains via Western blotting or in the ability of the Comp to reduce DTNB [18]. While the Comp strain does show more virulence *in vivo*, the mechanisms may be multifaceted and remain to be elucidated. However, it is evident that TrxA deficiency in *A*. *baumannii* has attenuated virulence during pulmonary infection. Overall pathology was not remarkable, a surprising find due to the acute nature and rapid mortality seen in the mice in the survival study, however, the RAC value was significantly higher in mice infected with ΔtrxA compared to WT and Comp indicating decreased pathology. In fact, the RAC value of mice challenged with the thioredoxin mutant was found to be similar to that of naïve mice.

Together, these results indicate that thioredoxin is a virulence factor as evidenced by decreased organ burdens, host response, and increased survival in mice infected with the ΔtrxA mutant. This further supports previous results from our lab showing that the thioredoxin-null mutant can act as a live vaccine in a sepsis model of infection. Further studies will assess the role of thioredoxin in maintaining cell surface hydrophilicity and the differential host response to changes in hydrophobicity.

## Supporting information

S1 Table List of top 50 dysregulated gene expression in *Acinetobacter baumannii* trxA mutant. (XLSX)

S2 Table Antimicrobial susceptibility. Bacteria were grown overnight at 37 ^o^C from frozen stock on a tryptic soy agar (TSA) slants. The drug susceptibility assay was ran on a BD Phoenix using a NMIC/ID-304 susceptibility panel. If available, maximum MIC is shown in μg/mL. R = resistant, I = intermediate.
(PDF)

S1 Fig The effect of temperature, salt concentration, and pH on growth of wild type (Ci79), trxA mutant, and complemented bacterial strains. Growth curve of bacteria grown in (A) varying temperatures or the absence and presence of (B) NaCl or (C) varying pH. Representative of 3 independent experiments, n = 6. a = significant between group 1&2, b = significant between groups 1&3, c = significant between groups 2&3. Statistical differences were determined by two-way ANOVA with Holm-Sidak correction (P < 0.05).
(PDF)

## References

[1] Antunes LC, Imperi F, Carattoli A, Visca P. Deciphering the multifactorial nature of Acinetobacter baumannii pathogenicity. PLoS One. 2011;6(8):e22674 Epub 2011/08/11. 10.1371/journal.pone.0022674 21829642 PMC3148234

[2] Clark NM, Zhanel GG, Lynch JP 3rd. Emergence of antimicrobial resistance among Acinetobacter species: a global threat. Curr Opin Crit Care. 2016;22(5):491–9. 10.1097/MCC.0000000000000337 . 27552304

[3] Fournier PE, Richet H. The epidemiology and control of Acinetobacter baumannii in health care facilities. Clin Infect Dis. 2006;42(5):692–9. Epub 2006/02/01. 10.1086/500202 . 16447117

[4] Dent L, Marshall D, Pratap S, Hulette R. Multidrug resistant Acinetobacter baumannii: a descriptive study in a city hospital. BMC Infectious Diseases. 2010;10(1):196 10.1186/1471-2334-10-196 20609238 PMC2909240

[5] Tacconelli E, Magrini N. Global priority list of antibiotic-resistant bacteria to guide research, discovery, and development of new antibiotics World Health Organization 2017;http://www.who.int/medicines/publications/global-priority-list-antibiotic-resistant-bacteria/en/.

[6] Joly-Guillou ML. Clinical impact and pathogenicity of *Acinetobacter*. Clin Microbiol Infect. 2005;11(11):868–73. 10.1111/j.1469-0691.2005.01227.x . 16216100

[7] van Faassen H, KuoLee R, Harris G, Zhao X, Conlan JW, Chen W. Neutrophils play an important role in host resistance to respiratory infection with *Acinetobacter baumannii* in mice. Infect Immun. 2007;75(12):5597–608. 10.1128/IAI.00762-07 17908807 PMC2168347

[8] Breslow JM, Monroy MA, Daly JM, Meissler JJ, Gaughan J, Adler MW, et al Morphine, but not trauma, sensitizes to systemic Acinetobacter baumannii infection. J Neuroimmune Pharmacol. 2011;6(4):551–65. 10.1007/s11481-011-9303-6 21826405 PMC3973428

[9] Breslow JM, Meissler JJ Jr., Hartzell RR, Spence PB, Truant A, Gaughan J, et al Innate immune responses to systemic *Acinetobacter baumannii* infection in mice: neutrophils, but not interleukin-17, mediate host resistance. Infect Immun. 2011;79(8):3317–27. 10.1128/IAI.00069-11 21576323 PMC3147579

[10] Wisplinghoff H, Bischoff T, Tallent SM, Seifert H, Wenzel RP, Edmond MB. Nosocomial bloodstream infections in US hospitals: analysis of 24,179 cases from a prospective nationwide surveillance study. Clin Infect Dis. 2004;39(3):309–17. Epub 2004/08/13. 10.1086/421946 . 15306996

[11] Young LS, Sabel AL, Price CS. Epidemiologic, clinical, and economic evaluation of an outbreak of clonal multidrug-resistant Acinetobacter baumannii infection in a surgical intensive care unit. Infect Control Hosp Epidemiol. 2007;28(11):1247–54. Epub 2007/10/11. 10.1086/521660 . 17926275

[12] Falagas ME, Rafailidis PI. Attributable mortality of Acinetobacter baumannii: no longer a controversial issue. Crit Care. 2007;11(3):134 Epub 2007/06/05. 10.1186/cc5911 17543135 PMC2206403

[13] Lu J, Holmgren A. The thioredoxin antioxidant system. Free Radic Biol Med. 2014;66:75–87. 10.1016/j.freeradbiomed.2013.07.036 . 23899494

[14] Cheng C, Dong Z, Han X, Wang H, Jiang L, Sun J, et al Thioredoxin A is essential for motility and contributes to host infection of *Listeria monocytogenes* via redox interactions. Front Cell Infect Microbiol. 2017;7:287 Epub 2017/07/14. 10.3389/fcimb.2017.00287 28702378 PMC5487381

[15] Windle HJ, Fox A, Ni Eidhin D, Kelleher D. The thioredoxin system of *Helicobacter pylori*. J Biol Chem. 2000;275(7):5081–9. 10.1074/jbc.275.7.5081 . 10671551

[16] Lin K, O'Brien KM, Trujillo C, Wang R, Wallach JB, Schnappinger D, et al *Mycobacterium tuberculosis* thioredoxin reductase is essential for thiol redox homeostasis but plays a minor role in antioxidant defense. PLoS Pathog. 2016;12(6):e1005675 Epub 2016/06/02. 10.1371/journal.ppat.1005675 27249779 PMC4889078

[17] Kraemer PS, Mitchell A, Pelletier MR, Gallagher LA, Wasnick M, Rohmer L, et al Genome-wide screen in *Francisella novicida* for genes required for pulmonary and systemic infection in mice. Infect Immun. 2009;77(1):232–44. Epub 2008/10/29. 10.1128/IAI.00978-08 18955478 PMC2612238

[18] Ketter PM, Yu JJ, Guentzel MN, May HC, Gupta R, Eppinger M, et al Acinetobacter baumannii gastrointestinal colonization is facilitated by secretory IgA which is reductively dissociated by bacterial thioredoxin A. mBio. 2018;9(4). Epub 2018/07/12. 10.1128/mBio.01298-18 29991584 PMC6050963

[19] Ainsworth S, Ketter PM, Yu JJ, Grimm RC, May HC, Cap AP, et al Vaccination with a live attenuated *Acinetobacter baumannii* deficient in thioredoxin provides protection against systemic *Acinetobacter* infection. Vaccine. 2017;35(26):3387–94. Epub 2017/05/20. 10.1016/j.vaccine.2017.05.017 28522011 PMC5510955

[20] Krueger F. Trim Galore!: A wrapper tool around Cutadapt and FastQC to consistently apply quality and adapter trimming to FastQ files. 2015.

[21] Kim D, Langmead B, Salzberg SL. HISAT: a fast spliced aligner with low memory requirements. Nat Methods. 2015;12(4):357–60. Epub 2015/03/10. 10.1038/nmeth.3317 25751142 PMC4655817

[22] Liao Y, Smyth GK, Shi W. featureCounts: an efficient general purpose program for assigning sequence reads to genomic features. Bioinformatics. 2014;30(7):923–30. Epub 2013/11/15. 10.1093/bioinformatics/btt656 . 24227677

[23] Robinson MD, McCarthy DJ, Smyth GK. edgeR: a Bioconductor package for differential expression analysis of digital gene expression data. Bioinformatics. 2010;26(1):139–40. Epub 2009/11/17. 10.1093/bioinformatics/btp616 19910308 PMC2796818

[24] Ketter P, Guentzel MN, Chambers JP, Jorgensen J, Murray CK, Cap AP, et al Genome Sequences of four *Acinetobacter baumannii*-*A*. *calcoaceticus* complex isolates from combat-related infections sustained in the Middle East. Genome Announc. 2014;2(1). Epub 2014/02/08. 10.1128/genomeA.00026-14 24503987 PMC3916481

[25] Haimes J, Kelley M. Demonstration of a ΔΔCq calculation method to compute relative gene expression from qPCR data. Thermo Scientific Tech Note. 2010;1.

[26] Wood CR, Ohneck EJ, Edelmann RE, Actis LA. A light-regulated type I pilus contributes to *Acinetobacter baumannii* biofilm, motility, and virulence functions. Infect Immun. 2018;86(9). Epub 2018/06/13. 10.1128/IAI.00442-18 29891547 PMC6105899

[27] O'Toole GA. Microtiter dish biofilm formation assay. J Vis Exp. 2011;(47). Epub 2011/02/11. 10.3791/2437 21307833 PMC3182663

[28] Nielsen TB, Yan J, Luna B, Spellberg B. Murine oropharyngeal aspiration model of ventilator-associated and hospital-acquired bacterial pneumonia. J Vis Exp. 2018;(136). Epub 2018/07/17. 10.3791/57672 30010650 PMC6102004

[29] Emery JL, Mithal A. The number of alveoli in the terminal respiratory unit of man during late intrauterine life and childhood. Arch Dis Child. 1960;35:544–7. Epub 1960/12/01. 10.1136/adc.35.184.544 13726619 PMC2012643

[30] Lanka GK, Yu JJ, Gong S, Gupta R, Mustafa SB, Murthy AK, et al IgA modulates respiratory dysfunction as a sequela to pulmonary chlamydial infection as neonates. Pathog Dis. 2016;74(3). Epub 2016/01/13. 10.1093/femspd/ftv121 26755533 PMC5975234

[31] Mattick JS. Type IV pili and twitching motility. Annu Rev Microbiol. 2002;56:289–314. Epub 2002/07/27. 10.1146/annurev.micro.56.012302.160938 . 12142488

[32] Cascales E, Christie PJ. The versatile bacterial type IV secretion systems. Nat Rev Microbiol. 2003;1(2):137–49. Epub 2004/03/24. 10.1038/nrmicro753 15035043 PMC3873781

[33] Leong CG, Bloomfield RA, Boyd CA, Dornbusch AJ, Lieber L, Liu F, et al The role of core and accessory type IV pilus genes in natural transformation and twitching motility in the bacterium Acinetobacter baylyi. PLoS One. 2017;12(8):e0182139 Epub 2017/08/05. 10.1371/journal.pone.0182139 28771515 PMC5542475

[34] Sun D, Crowell SA, Harding CM, De Silva PM, Harrison A, Fernando DM, et al KatG and KatE confer *Acinetobacter* resistance to hydrogen peroxide but sensitize bacteria to killing by phagocytic respiratory burst. Life Sci. 2016;148:31–40. Epub 2016/02/11. 10.1016/j.lfs.2016.02.015 26860891 PMC4792659

[35] Vila J, Pachon J. Therapeutic options for Acinetobacter baumannii infections. Expert Opin Pharmacother. 2008;9(4):587–99. Epub 2008/03/04. 10.1517/14656566.9.4.587 . 18312160

[36] Latibeaudiere R, Rosa R, Laowansiri P, Arheart K, Namias N, Munoz-Price LS. Surveillance cultures growing carbapenem-resistant *Acinetobacter baumannii* predict the development of clinical infections: A retrospective cohort study. Clinical Infectious Diseases. 2015;60(3):415–22. 10.1093/cid/ciu847 25352586

[37] Manchanda V, Sanchaita S, Singh N. Multidrug resistant *Acinetobacter*. Journal of Global Infectious Diseases. 2010;2(3):291–304. 10.4103/0974-777X.68538 20927292 PMC2946687

[38] Laurent TC, Moore EC, Reichard P. Enzymatic synthesis of deoxyribonucleotides. Iv. Isolation and characterization of thioredoxin, the hydrogen donor from *Escherichia coli* B. J Biol Chem. 1964;239:3436–44. . 14245400

[39] Miranda-Vizuete A, Damdimopoulos AE, Gustafsson J, Spyrou G. Cloning, expression, and characterization of a novel *Escherichia coli* thioredoxin. J Biol Chem. 1997;272(49):30841–7. 10.1074/jbc.272.49.30841 . 9388228

[40] Stewart EJ, Aslund F, Beckwith J. Disulfide bond formation in the *Escherichia coli* cytoplasm: an in vivo role reversal for the thioredoxins. EMBO J. 1998;17(19):5543–50. 10.1093/emboj/17.19.5543 9755155 PMC1170883

[41] Heindorf M, Kadari M, Heider C, Skiebe E, Wilharm G. Impact of Acinetobacter baumannii superoxide dismutase on motility, virulence, oxidative stress resistance and susceptibility to antibiotics. PLoS One. 2014;9(7):e101033 10.1371/journal.pone.0101033 25000585 PMC4085030

[42] Reardon‐Robinson ME, Osipiuk J, Jooya N, Chang C, Joachimiak A, Das A, et al A thiol‐disulfide oxidoreductase of the Gram-positive pathogen *Corynebacterium diphtheriae* is essential for viability, pilus assembly, toxin production and virulence. Molecular microbiology. 2015;98(6):1037–50. 10.1111/mmi.13172 26294390 PMC4981772

[43] Ambalam P, Kondepudi KK, Nilsson I, Wadstrom T, Ljungh A. Bile stimulates cell surface hydrophobicity, Congo red binding and biofilm formation of *Lactobacillus* strains. FEMS Microbiol Lett. 2012;333(1):10–9. Epub 2012/05/09. 10.1111/j.1574-6968.2012.02590.x . 22563647

