# Supplementary material for: Thioredoxin-A is a virulence factor and mediator of the type IV pilus system in Acinetobacter baumannii
Source: PLoS One. 2019 Jul 2;14(7):e0218505. doi: 10.1371/journal.pone.0218505 (PMC6605650; doi:10.1371/journal.pone.0218505)
Supplement: S2 Table — Bacteria were grown overnight at 37 oC from frozen stock on a tryptic soy agar (TSA) slants. The drug susceptibility assay was ran on a BD Phoenix using a NMIC/ID-304 susceptibility panel. If available, maximum MIC is shown in μg/mL. R = resistant, I = intermediate. (PDF) [file pone.0218505.s002.pdf]

	Ci79 (WT)	ΔtrxA	Comp
**Amikacin**	>32 R	>32 R	>32 R
**Amoxicillin-Clavulanate**	R	R	R
**Ampicillin**	R	R	R
**Aztreonam**	8 R	>16 R	>16 R
**Cefazolin**	R	R	R
**Cefepime**	16 I	>16 R	>16 R
**Ceftazidime**	16 I	>16 R	16 I
**Ceftriaxone**	>32 R	>32 R	>32 R
**Ciprofloxacin**	>2 R	>2 R	>2 R
**Ertapenem**	R	R	R
**Gentamicin**	>8 R	>8 R	>8 R
**Imipenem**	>8 R	>8 R	>8 R
**Levofloxacin**	>4 R	>4 R	>4 R
**Nitrofurantoin**	R	R	R
**Tetracycline**	>8 R	>8 R	>8 R
**Tobramycin**	>8 R	>8 R	>8 R

Supplemental 2 Table. Antimicrobial susceptibility. Bacteria were grown overnight at 37 ^{o}C from frozen stock on a tryptic soy agar (TSA) slants. The drug susceptibility assay was ran on a BD Phoenix using a NMIC/ID-304 susceptibility panel. If available, maximum MIC is shown in µg/mL.
R= resistant, I = intermediate.
