# Supplementary material for: Thioredoxin-A is a virulence factor and mediator of the type IV pilus system in Acinetobacter baumannii
Source: PLoS One. 2019 Jul 2;14(7):e0218505. doi: 10.1371/journal.pone.0218505 (PMC6605650; doi:10.1371/journal.pone.0218505)
Supplement: S1 Fig — Growth curve of bacteria grown in (A) varying temperatures or the absence and presence of (B) NaCl or (C) varying pH. Representative of 3 independent experiments, n = 6. a = significant between group 1&2, b = significant between groups 1&3, c = significant between groups 2&3. Statistical differences were determined by two-way ANOVA with Holm-Sidak correction (P < 0.05). (PDF) [file pone.0218505.s003.pdf]

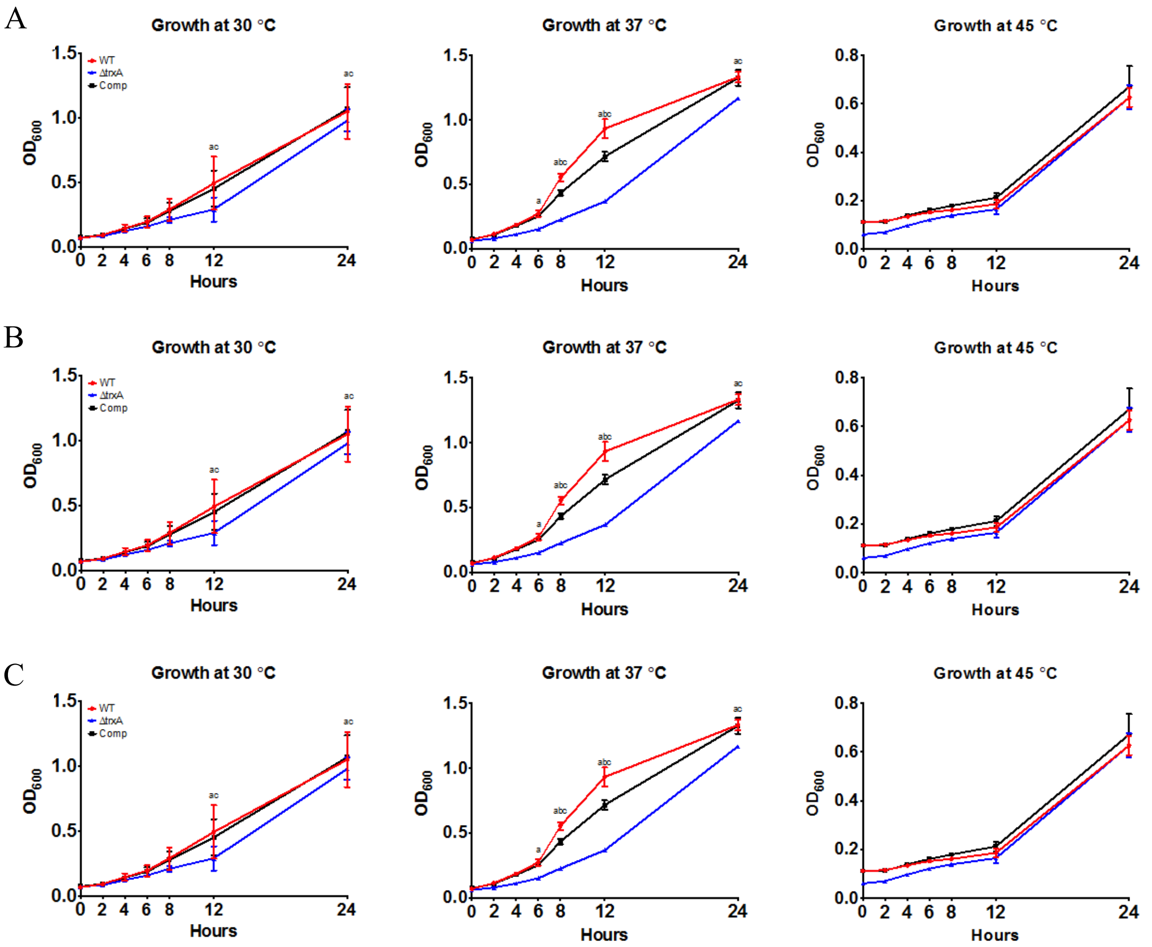

Supplemental 1 Fig. The effect of temperature, salt concentration, and pH on growth of wild type (Ci79), trxA mutant, and complemented bacterial strains. Growth curve of bacteria grown in (A) varying temperatures or the absence and presence of (B) NaCl or (C) varying pH. Representative of 3 independent experiments, N=6. a = significant between group 1&2, b = significant between groups 1&3, c = significant between groups 2&3. Statistical differences were determined by two-way ANOVA with Holm-Sidak correction ($P < 0.05$).
